# Plasminogen Activator Inhibitor-1 Is Involved in Impaired Bone Repair Associated with Diabetes in Female Mice

**DOI:** 10.1371/journal.pone.0092686

**Published:** 2014-03-20

**Authors:** Li Mao, Naoyuki Kawao, Yukinori Tamura, Katsumi Okumoto, Kiyotaka Okada, Masato Yano, Osamu Matsuo, Hiroshi Kaji

**Affiliations:** 1 Department of Physiology and Regenerative Medicine, Kinki University Faculty of Medicine, Osaka, Japan; 2 Life Science Research Institute, Kinki University, Osaka, Japan; INSERM U1059/LBTO, Université Jean Monnet, France

## Abstract

Previous studies suggest that fracture healing is impaired in diabetes; however, the underlying mechanism remains unclear. Here, we investigated the roles of plasminogen activator inhibitor-1 (PAI-1) in the impaired bone repair process by using streptozotocin (STZ)-induced diabetic female wild-type (*PAI-1* ^+/+^) and PAI-1-deficient (*PAI-1* ^−/−^) mice. Bone repair and the number of alkaline phosphatase (ALP)-positive cells at the site of a femoral bone damage were comparable in *PAI-1* ^+/+^ and *PAI-1* ^−/−^ mice without STZ treatment. Although the bone repair process was delayed by STZ treatment in *PAI-1* ^+/+^ mice, this delayed bone repair was blunted in *PAI-1* ^−/−^ mice. The reduction in the number of ALP-positive cells at the site of bone damage induced by STZ treatment was attenuated in *PAI-1* ^−/−^ mice compared to *PAI-1* ^+/+^ mice. On the other hand, PAI-1 deficiency increased the levels of ALP and type I collagen mRNA in female mice with or without STZ treatment, and the levels of Osterix and osteocalcin mRNA, suppressed by diabetic state in *PAI-1* ^+/+^ mice, were partially protected in *PAI-1* ^−/−^ mice. PAI-1 deficiency did not affect formation of the cartilage matrix and the levels of types II and X collagen and aggrecan mRNA suppressed by STZ treatment, although PAI-1 deficiency increased the expression of chondrogenic markers in mice without STZ treatment. The present study indicates that PAI-1 is involved in the impaired bone repair process induced by the diabetic state in part through a decrease in the number of ALP-positive cells.

## Introduction

The risk of fracture is increased in diabetic patients mainly due to impairment of the bone formation process [1], [2]. It is well accepted that type 1 diabetes is associated with a significant decrease in skeletal mass and an increase in fracture risk [1], [2]. The detrimental skeletal effects of glucose toxicity, insulin deficiency and diabetic complications might partly explain the association between type 1 diabetes and osteoporosis [3]–[5]. Previous findings suggest that a decrease in osteoblastic bone formation is a major contributor to diabetic osteoporosis [4], [6]. However, the pathogenesis of this skeletal fragility and impaired bone formation in type 1 diabetic patients remain to be fully clarified.

The recruitment of bone marrow-derived mesenchymal stem cells to the site of injury is impaired in diabetic patients [7]. Impaired mobilization and a reduced number of endothelial progenitor cells in diabetic patients prevent vasculogenesis and delay the healing process [8]. Cells that are critical to wound healing, such as fibroblasts, keratinocytes, and endothelial cells, become functionally impaired when exposed to a diabetic high-glucose environment *in vitro* [9], [10]. In human studies, hematopoietic stem cell and proangiogenic cell mobilization in response to granulocyte colony-stimulating factor is impaired in diabetes, which might be related to the impaired tissue repair [11]. Several studies indicate that diabetes leads to impaired fracture healing [12]–[16], and this abnormal repair is insulin-dependent, because it was reversed by insulin treatment [13], [17]. More recent studies have shown that the impaired fracture healing observed in diabetic state might be due to the loss of cartilage caused by increased apoptosis and osteoclastogenesis, which is followed by a reduction in the cartilaginous template for endochondral bone formation [13]–[15]. Increased chondrocyte and osteoblast apoptosis is tumor necrosis factor (TNF)-α-mediated, along with increased osteoclast survival, leading to destruction of early callus tissue and impeding fracture healing [13]–[15]. However, the details in mechanism of impaired bone repair and fracture healing in diabetic state still remain unclear.

Plasminogen activator inhibitor-1 (PAI-1) is the principal inhibitor of plasminogen activator, and hence fibrinolysis. Several reports have shown that circulating PAI-1 levels are elevated in type 1 and type 2 diabetic patients and animals [18]–[20]. PAI-1 has various functions, including regulation of extracellular matrix (ECM) degradation, cell migration, and apoptosis [21]. Daci *et al*. previously reported that PAI-1 deficiency partially protects against bone loss in estrogen-deficient mice [22]. Our recent study also showed that PAI-1 deficiency protects against diabetic bone loss in female mice [20]. Clarifying the mechanism of bone repair and regeneration is important to meet the clinical demand for bone reconstruction. We recently showed that plasminogen is crucial for bone repair in mice [23], indicating that the tissue fibrinolytic system is important for bone repair and fracture healing. These findings suggest that PAI-1 may play a crucial role in the bone repair process. However, the role of PAI-1 in impaired bone repair process observed in diabetes is unknown.

In the present study, we therefore examined the effects of PAI-1 deficiency on the impaired bone repair in diabetes by using a mouse model of diabetes induced by streptozotocin (STZ) in female wild-type (*PAI-1* ^+/+^) and PAI-1- deficient (*PAI-1* ^−/−^) mice.

## Materials and Methods

### Ethics statement

All mouse experiments were performed according to the Guide for the Care and Use of Laboratory Animals from the National Institutes of Health and the institutional guidelines for the use and care of laboratory animals at Kinki University. The protocol was approved by the Experimental Animal Welfare Committee of Kinki University (permit numbers: KAME-23-023 and KAME-24-009). All surgeries and the collection of quantitative computed tomography (qCT) images were performed under 2% isoflurane. The mice were euthanized with pentobarbital sodium (over 50 mg/kg, intraperitoneally). All efforts were made to minimize suffering.

### Animals

Female *PAI-1* ^+/+^ and *PAI-1* ^−/−^ mice, 10 weeks old, with a mixed C57BL/6J (81.25%) and 129/SvJ (18.75%) background, were used. These mice were kindly provided by Professor D. Collen (University of Leuven, Belgium).

### Diabetic mouse model

Diabetes was induced in female *PAI-1* ^+/+^ and *PAI-1* ^−/−^ mice by daily intraperitoneal injections of STZ (50 mg/kg body weight, in saline) (Sigma, St Louis, MO, USA, # S0130), a pancreatic β-cell cytotoxin, for 4 days, as previously described [20]. Controls were injected with saline. Four days after the last injection (day 4), non-fasting blood glucose levels were measured with a glucometer (Glutest Ace, Sanwa Kagaku Kenkyusyo, Nagoya, Japan) by using blood obtained from the tail vein. Mice with blood glucose levels greater than 300 mg/dl were considered diabetic. At 2 weeks after induction of diabetes, a bone defect surgery was performed in the right femur of the mice. Animals were maintained in metabolic cages on a 12-h light/dark cycle, and they received food and water *ad libitum*.

### Blood measurements

Blood was obtained from mice at 4 weeks after induction of diabetes. Plasma total PAI-1 was measured using a Murine Total PAI-1 ELISA kit (Molecular Innovations, MI, USA, # MPAIKT-TOT).

### Bone defect model

A bone defect was induced in the mice according to a previously described method [23]. Briefly, under anesthesia induced by 2% isoflurane, the anterior skin over the mid-femur of the right leg was incised longitudinally for 5 mm in length. After splitting the muscle, the surface of femoral bone was exposed. Then, a hole was made using a drill with a diameter of 0.9 mm during the saline irrigation to prevent thermal necrosis of the margins. The incised skin was then sutured in a sterile manner, and anesthesia was discontinued. Body temperature was maintained at 37°C during surgery by a heating pad.

### *In vivo* quantitative computed tomography (qCT) analysis

The mice were anesthetized using 2% isoflurane, and the femur was scanned using an X-ray CT system (Latheta LCT-200; Hitachi Aloka Medical, Tokyo, Japan). Parameters for the CT scans were as follows: tube voltage, 50 kVp; tube current, 500 μA; integration time, 3.6 ms; axial field of view, 48 mm, with an isotropic voxel size of 48 μm. Images were generated by integration of 2 signal averages for the femur. Total scan time was approximately 5 min. Volume-rendered 3-dimensional CT pictures were reconstructed using the VGStudio MAX2.2 software (Nihon Visual Science, Tokyo, Japan). The area of the bone defect in the femur was quantified using an image processing program (ImageJ, http://rsbweb.nih.gov/ij/download.html).

### Histological analysis

Mice were anesthetized using pentobarbital sodium (50 mg/kg, intraperitoneally) on day 7 after surgery. The femur was removed, fixed in 4% paraformaldehyde, demineralized in 22.5% formic acid and 340 mM sodium citrate solution for 24 h, and then embedded in paraffin. Thereafter, 4-μm-thick sections were obtained. Immunostaining was performed as described previously [23]. Briefly, the sections were incubated with an anti-alkaline phosphatase (ALP) antibody (Abnova, Taipei, Taiwan, # PAB12279) at a dilution of 1∶100 followed by incubation with the appropriate secondary antibody conjugated with horseradish peroxidase (Nichirei Biosciences Inc., Tokyo, Japan, # 414341). Positive signals were visualized using a tyramide signal amplification system (PerkinElmer, Waltham, MS, USA, # NEL744B001KT). The number of ALP-positive cells per 0.1 mm^2^ in the microscopic fields of the damaged site of femur was quantified in a blinded evaluation, as described previously [23]. The number of ALP-positive osteoblast-like cells per 1 mm of bone surface (N.Ob/BS) and osteoblast surface per bone surface (Ob.S/BS) were measured according to the guidelines of the American Society of Bone and Mineral Research [24]. The sections were stained with tartrate-resistant acid phosphatase (TRAP) by using a TRAP staining kit (Wako Pure Chem., Osaka, Japan, # 294-678001). The number of TRAP-positive multinucleated cells per 1 mm of bone surface was measured at the damaged site of the femur in a blinded evaluation. The sections were processed for Alcian blue and toluidine blue staining. The areas of the cartilage matrices that included proteoglycans and glycosaminoglycans were quantified by measuring the Alcian blue-positive areas and the metachromatic areas in the sections stained with toluidine blue using image processing software (Mac SCOPE; Mitani Co., Fukui, Japan) in a blinded evaluation.

### Real-time polymerase chain reaction (PCR) analysis

Bone samples were crushed in liquid nitrogen, and total RNA was extracted from the homogenized samples using an RNeasy mini kit (Qiagen, Tokyo, Japan, # 74104). Real-time PCR was performed with a StepOne Plus cycler using Fast SYBR GREEN PCR Master Mix (Life Technologies Japan, Tokyo, Japan, # 4385610). The primer sets used are shown in Table S1. The mRNA levels of target genes in the mouse tissues were normalized relative to the levels of glyceraldehyde-3-phosphate dehydrogenase (GAPDH) mRNA.

### Statistical analysis

Data are expressed as means ± SEM. Statistical significance was assessed using unpaired 2-tailed *t*-tests and one-way ANOVA. Differences with *p* values less than 0.05 were considered statistically significant. All statistical analyses were performed using StatView version 5.0 software (SAS Institute; Cary, NC, USA).

## Results

### Effects of STZ treatment in female mice

**Figure 1:**
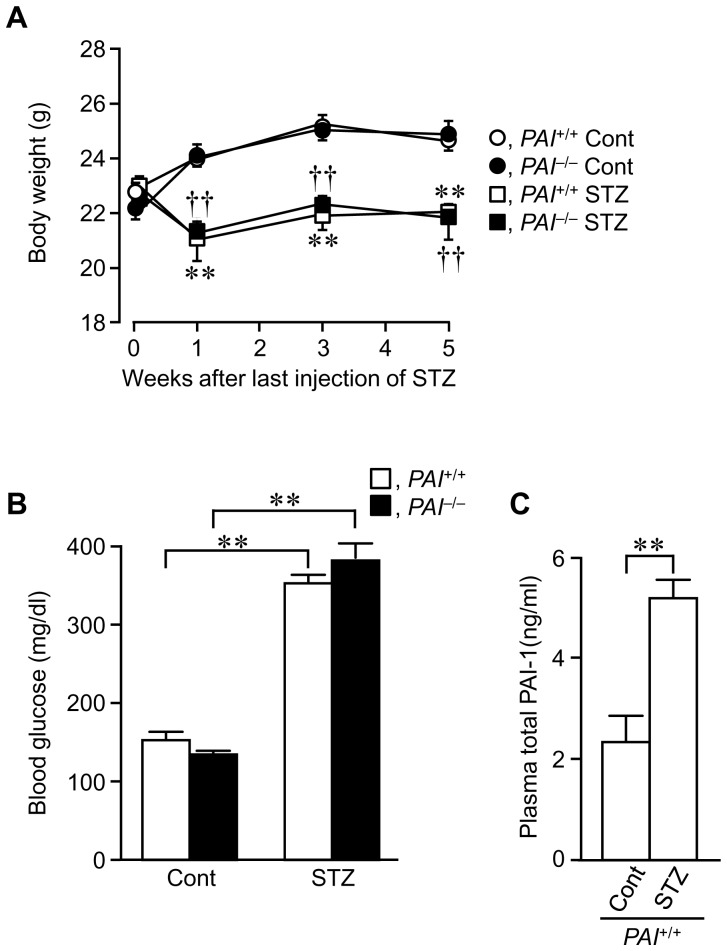
Effects of streptozotocin (STZ) treatment on body weight, blood glucose, and plasma PAI-1 levels in female mice. (A) Growth curve during experiments in control (Cont) and STZ-treated female *PAI-1* ^+/+^ and *PAI-1* ^−/−^ mice. Results are expressed as the means ± SEM. **p<0.01 vs. the *PAI-1* ^+/+^ control group, ^††^ *p*<0.01 vs. *PAI-1* ^−/−^ control group. (n = 5 in each group). (B) Blood glucose in control and STZ-treated female *PAI-1* ^+/+^ and *PAI-1* ^−/−^ mice. Results are expressed as means ± SEM. **p<0.01 (n = 5 in each group). (C) Plasma total PAI-1 levels in control and STZ-treated female *PAI-1* ^+/+^ mice. Results are expressed as means ± SEM. **p<0.01 (n = 5 in each group).

STZ treatment decreased the body weight of *PAI* ^+/+^ and *PAI* ^−/−^ mice from 7 days after the last injection of STZ (Fig. 1A). Four days after the final STZ injection, blood glucose levels were markedly elevated in *PAI* ^+/+^ and *PAI* ^−/−^ mice (Fig. 1B), indicating that STZ induced diabetes in the mice. Consistent with the elevation in blood glucose levels, circulating PAI-1 levels were elevated by STZ treatment in *PAI* ^+/+^ mice (Fig. 1C). These data were compatible with those obtained in our previous study [20].

### Bone repair after a femoral bone defect

**Figure 2:**
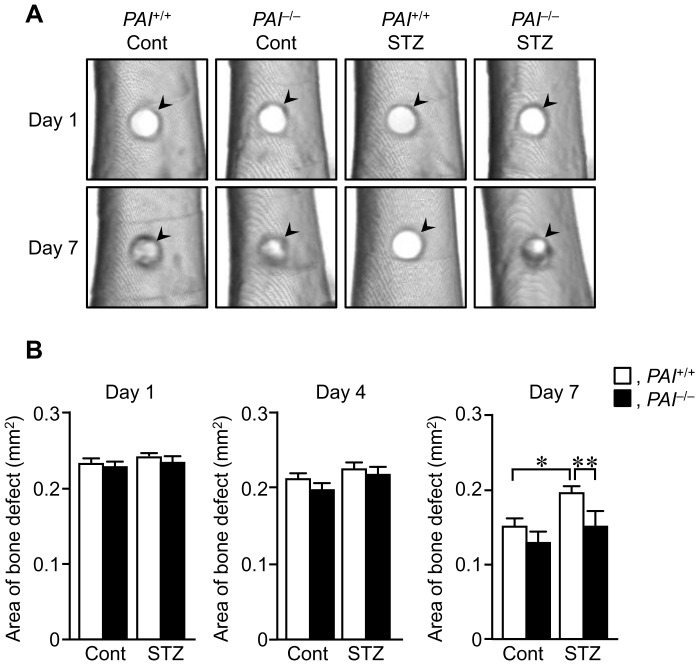
Bone repair after a femoral bone defect. (A) Three-dimensional images of the damaged site on days 1 and 7 after a femoral bone defect in female *PAI-1* ^+/+^ and *PAI-1* ^−/−^ mice with or without STZ treatment, as assessed by qCT. The arrowheads indicate the damaged site. (B) Quantification of the area at the damaged site on days 1, 4, and 7 after a femoral bone defect in female *PAI-1* ^+/+^ and *PAI-1* ^−/−^ mice with or without STZ treatment. Data represent the mean ± SEM of 5-6 mice. *p<0.05, **p<0.01. Cont, control.

The damaged site on the femur was progressively and similarly repaired in *PAI-1* ^+/+^ and *PAI-1* ^−/−^ mice without STZ treatment until day 7, as assessed by qCT (Fig. 2A, B). Conversely, the damaged site significantly remained on day 7 in *PAI-1* ^+/+^ mice with STZ treatment (Fig. 2A, B). This delay in bone repair was blunted in *PAI-1* ^−/−^ mice with STZ treatment.

### Histological analysis of the damaged site after a femoral bone defect

**Figure 3:**
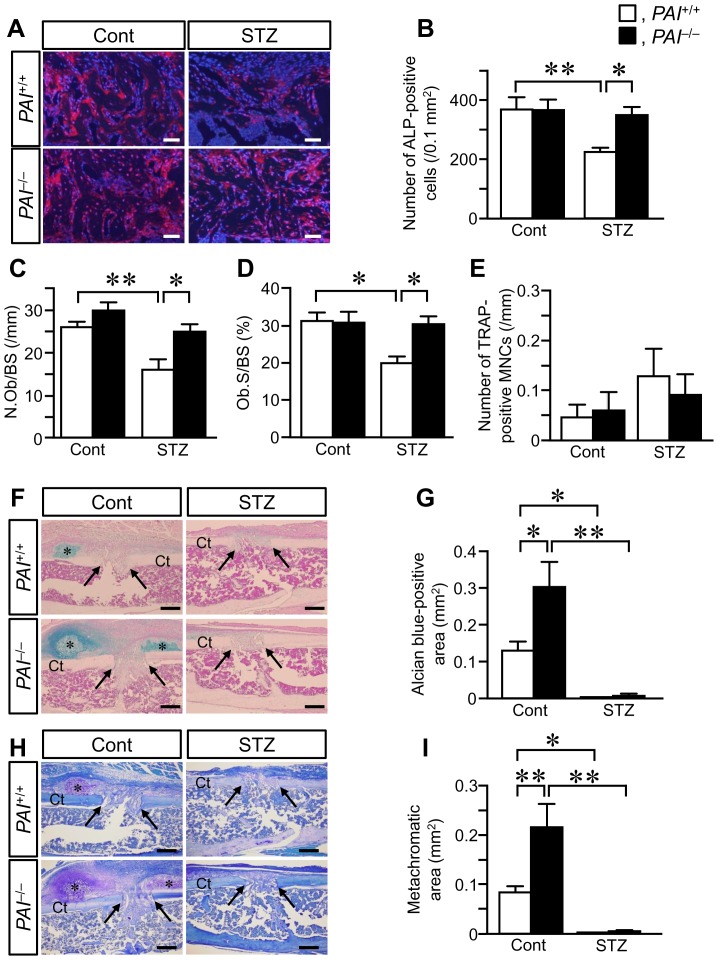
Histological analysis of the damaged site after a femoral bone defect. (A) Microphotographs of ALP-positive cells at the damaged site on day 7 after a femoral bone defect in female *PAI-1* ^+/+^ and *PAI-1* ^−/−^ mice with or without STZ treatment. (B) The number of ALP-positive cells per 0.1 mm^2^ of the microscopic field in the damaged site on day 7 after a femoral bone defect in female *PAI-1* ^+/+^ and *PAI-1* ^−/−^ mice with or without STZ treatment. (C) The number of ALP-positive osteoblast-like cells per 1 mm of bone surface (N.Ob/BS) at the damaged site on day 7 in female *PAI-1* ^+/+^ and *PAI-1* ^−/−^ mice with or without STZ treatment. (D) Osteoblast surface per bone surface (Ob.S/BS) at the damaged site on day 7 in female *PAI-1* ^+/+^ and *PAI-1* ^−/−^ mice with or without STZ treatment. (E) The number of TRAP-positive multinucleated cells (MNCs) per 1 mm of bone surface at the damaged site on day 7 in female *PAI-1* ^+/+^ and *PAI-1* ^−/−^ mice with or without STZ treatment. (F) Microphotographs of Alcian blue-stained sections at the damaged site on day 7 after a femoral bone defect in female *PAI-1* ^+/+^ and *PAI-1* ^−/−^ mice with or without STZ treatment. The sections were counterstained with nuclear fast red. Asterisks indicate the Alcian blue-positive region. (G) Quantification of the Alcian blue-positive area at the damaged site on day 7 in female *PAI-1* ^+/+^ and *PAI-1* ^−/−^ mice with or without STZ treatment. (H) Microphotographs of toluidine blue-stained sections at the damaged site on day 7 after a femoral bone defect in female *PAI-1* ^+/+^ and *PAI-1* ^−/−^ mice with or without STZ treatment. Asterisks indicate the metachromatic region. (I) Quantification of the area of the metachromatic region in the sections stained with toluidine blue at the damaged site on day 7 in female *PAI-1* ^+/+^ and *PAI-1* ^−/−^ mice with or without STZ treatment. The results are from experiments performed on 5-6 mice in each group (A, F, H). Scale bars indicate 50 (A) and 300 μm (F, H). The arrows indicate the edge of the damaged site in the inner cortex (F, H). Data represent the mean ± SEM of 5-6 mice (B-E, G, I). *p<0.05, **p<0.01. Cont, control. Ct, cortical bone.

The number of ALP-positive cells at the damaged site on day 7 was similar in both *PAI-1* ^+/+^ and *PAI-1* ^−/−^ mice without STZ treatment (Fig. 3A, B). STZ treatment significantly reduced the number of ALP-positive cells at the damaged site in female *PAI-1* ^+/+^ mice (Fig. 3A, B). However, consistent with the decreased bone repair observed in the diabetic state, PAI-1 deficiency significantly blunted the reduction in the number of ALP-positive cells at the damaged site in diabetic *PAI-1* ^−/−^ mice (Fig. 3A, B). N.Ob/BS and Ob.S/BS at the damaged site were similar on day 7 in *PAI-1* ^+/+^ and *PAI-1* ^−/−^ mice without STZ treatment (Fig. 3C, D). Although STZ treatment significantly reduced the N.Ob/BS and Ob.S/BS at the damaged site in female *PAI-1* ^+/+^ mice, PAI-1 deficiency significantly blunted the reductions in N.Ob/BS and Ob.S/BS induced by the diabetic state (Fig. 3C, D). Conversely, the number of TRAP-positive multinucleated cells per bone surface was not significantly different between *PAI-1* ^+/+^ and *PAI-1* ^−/−^ mice on day 7 regardless of STZ treatment (Fig. 3E).

PAI-1 deficiency significantly increased the formation of the cartilage matrix at the damaged site on day 7 after a femoral bone defect in sections stained with Alcian blue and toluidine blue (Fig. 3F–I). STZ treatment significantly reduced the formation of cartilage matrix in *PAI-1* ^+/+^ and *PAI-1* ^−/−^ mice. PAI-1 deficiency did not affect the formation of cartilage matrix suppressed by STZ (Fig. 3F–I).

### Analysis of osteogenic, bone resorption, osteoclast, chondrogenic, and adipogenic marker gene expression in bone tissues at the damaged site

**Figure 4:**
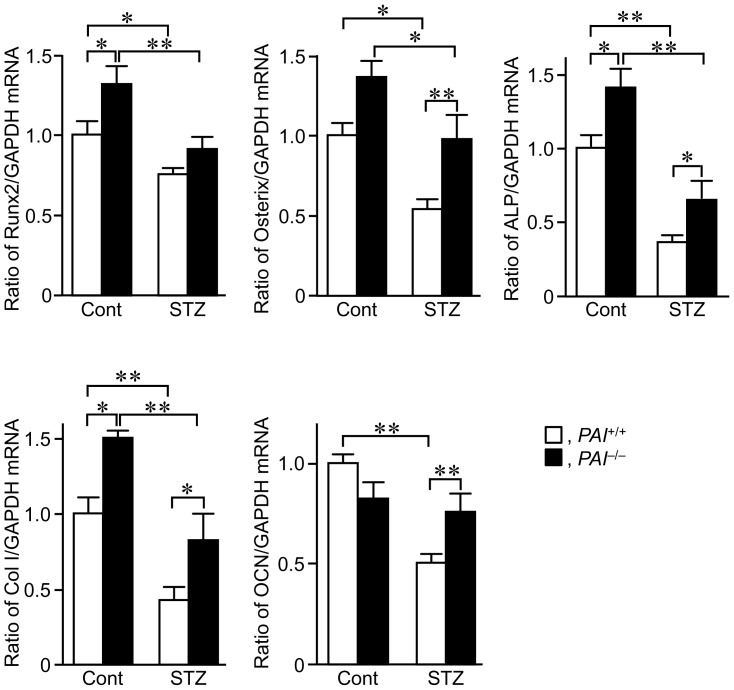
Effects of PAI-1 deficiency on osteogenic differentiation during bone repair in female mice. The levels of Runx2, Osterix, ALP, type I collagen (Col I), and osteocalcin (OCN) mRNA at the damaged site on day 7 after a femoral bone defect in female *PAI-1* ^+/+^ and *PAI-1* ^−/−^ mice with or without STZ treatment. Data are expressed relative to GAPDH mRNA values, and are expressed as the mean ± SEM. *p<0.05, **p<0.01 (n = 5–7 in each group). Cont, control.

Next, we examined the levels of osteogenic marker mRNA in the bone tissues at the damaged site after a femoral bone defect in female *PAI-1* ^+/+^ and *PAI-1* ^−/−^ mice. The levels of Runx2, ALP, and type I collagen mRNA were significantly increased in *PAI-1* ^−/−^ mice, compared to those in *PAI-1* ^+/+^ mice without STZ treatment (Fig. 4). The levels of Osterix and osteocalcin mRNA were similar in *PAI-1* ^+/+^ and *PAI-1* ^−/−^ mice without STZ treatment on day 7 (Fig. 4). STZ treatment significantly reduced the levels of Runx2, Osterix, ALP, type I collagen, and osteocalcin mRNA in *PAI-1* ^+/+^ mice, and PAI-1 deficiency significantly blunted the levels of Osterix, ALP, type I collagen, and osteocalcin mRNA that were suppressed by STZ treatment. (Fig. 4).

**Figure 5:**
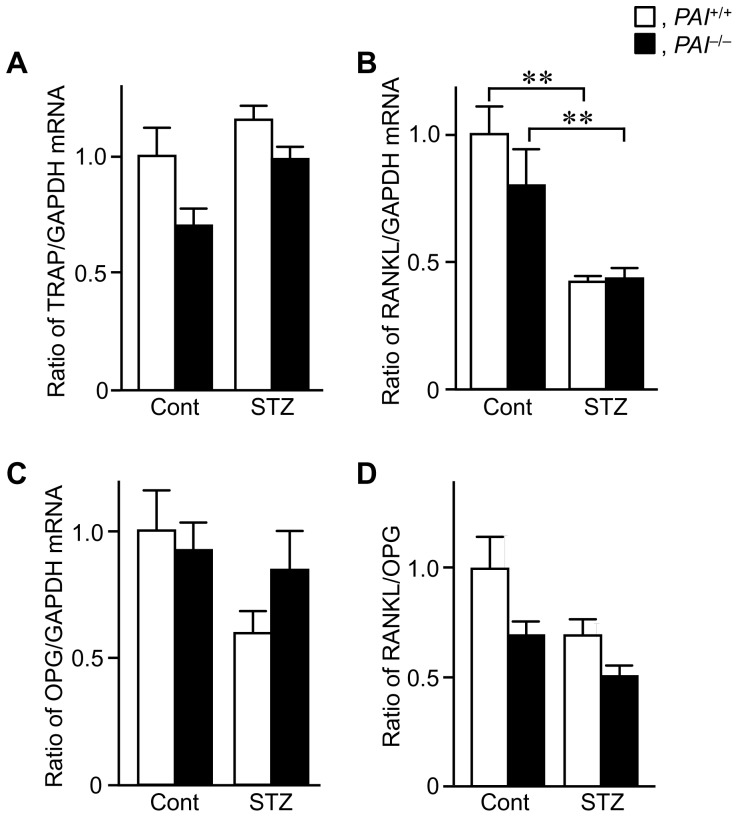
Effects of PAI-1 deficiency on bone resorption markers during bone repair in female mice. (A–C) The levels of TRAP, RANKL, and osteoprotegerin (OPG) mRNA at the damaged site on day 7 after a femoral bone defect in female *PAI-1* ^+/+^ and *PAI-1* ^−/−^ mice with or without STZ treatment. Data are expressed relative to GAPDH mRNA values, and are expressed as mean ± SEM. (D) The ratio of RANKL/OPG mRNA at the damaged site on day 7 after a femoral bone defect in female *PAI-1* ^+/+^ and *PAI-1* ^−/−^ mice with or without STZ treatment. **p<0.01 (n = 5–7 in each group). Cont, control.

We examined the levels of bone resorption markers in bone tissues at the damaged site after a femoral bone defect in female mice. The levels of TRAP, receptor activator of nuclear factor κB ligand (RANKL), and osteoprotegerin (OPG) mRNA as well as the ratio of RANKL/OPG were similar in *PAI-1* ^+/+^ and *PAI-1* ^−/−^ mice without STZ treatment on day 7 (Fig. 5A–D). STZ treatment significantly reduced the levels of RANKL mRNA in *PAI-1* ^+/+^ and *PAI-1* ^−/−^ mice (Fig. 5B), although it did not affect the levels of TRAP mRNA (Fig. 5A). STZ treatment seemed to reduce the levels of OPG mRNA and the ratio of RANKL/OPG in *PAI-1* ^+/+^ mice, although the differences were not statistically significant (Fig. 5C, D). PAI-1 deficiency did not affect the levels of RANKL mRNA suppressed by STZ treatment (Fig. 5B).

**Figure 6:**
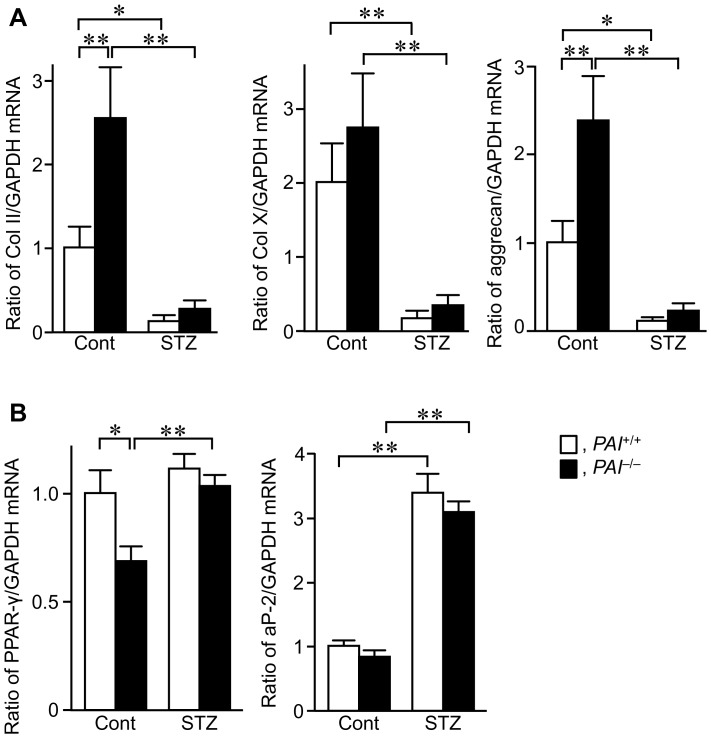
Effects of PAI-1 deficiency on chondrogenic or adipogenic differentiation during bone repair in female mice. (A) The levels of type II collagen (Col II), type X collagen (Col X), and aggrecan mRNA at the damaged site on day 7 after a femoral bone defect in female *PAI-1* ^+/+^ and *PAI-1* ^−/−^ mice with or without STZ treatment. Data are expressed relative to GAPDH mRNA values, and are expressed as mean ± SEM. *p<0.05, **p<0.01 (n = 5–7 in each group). (B) The levels of PPAR-γ and aP-2 mRNA at the damaged site on day 7 after a femoral bone defect in female *PAI-1* ^+/+^ and *PAI-1* ^−/−^ mice with or without STZ treatment. Data are expressed relative to GAPDH mRNA values, and are expressed as mean ± SEM. *p<0.05, **p<0.01 (n = 5–7 in each group). Cont, control.

Finally, we examined the levels of chondrogenic and adipogenic markers in bone tissues at the damaged site after a femoral bone defect in female *PAI-1* ^+/+^ and *PAI-1* ^−/−^ mice. As shown in Fig. 6A, PAI-1 deficiency significantly enhanced the levels of type II collagen and aggrecan mRNA in bone tissues at the damaged site after a femoral bone defect, although it did not affect the levels of type X collagen mRNA. STZ treatment significantly reduced the levels of chondrogenic markers in *PAI-1* ^+/+^ mice, and PAI-1 deficiency did not affect the levels of chondrogenic markers suppressed by STZ treatment (Fig. 6A). In contrast, PAI-1 deficiency decreased the levels of peroxisome proliferator-activated receptor γ (PPAR-γ) mRNA, but not adipocyte protein-2 (aP-2) mRNA at the damaged site after a femoral bone defect (Fig. 6B). STZ treatment enhanced the levels of aP-2 mRNA, but not PPAR-γ mRNA in *PAI-1* ^+/+^ mice. PAI-1 deficiency did not affect the levels of adipogenic marker genes in mice with STZ treatment (Fig. 6B).

## Discussion

PAI-1, as a pivotal component of fibrinolytic system, belongs to the serine protease inhibitor (serpin) superfamily [25]. It is synthesized in several tissues, including bone, and is the principal inhibitor of plasminogen activators (PA) [26]. The fibrinolytic system participates in a wide variety of functions in mammals beyond its proteolytic effect on thrombi. Several studies have suggested the involvement of the PA/PAI-1 system in bone metabolism [27], [28]. Our recent study using plasminogen-deficient mice showed that plasminogen deficiency decreases cartilage formation and osteoblastogenesis at the damaged site of femur after bone defect [23]. These findings imply that the tissue fibrinolytic system is involved in the bone repair process.

PAI-1 deficiency protects from increased bone turnover and trabecular bone loss in pathological states, such as estrogen deficiency [22]. Moreover, we recently demonstrated that PAI-1 deficiency protects against diabetic bone loss in female mice [20]. These observations suggest a specific role for PAI-1 in the regulation of trabecular bone turnover and osteoblastic bone formation, especially in some specific pathological conditions. The present study showed that diabetic wild-type mice displayed an obvious delay in bone repair, compared to non-diabetic wild-type mice from qCT analysis, which was compatible with previous evidence on fracture repair [16]. Moreover, PAI-1 deficiency rescued the impaired bone repair caused by the diabetic state, indicating that PAI-1 is involved in the delayed bone repair induced by the diabetic state in female mice. Our previous study suggested that the diabetic state increases PAI-1 expression in the liver, resulting in elevation of circulating PAI-1 levels in female mice [20]. The increased levels of circulating PAI-1 induced by the diabetic state might be responsible for the impaired bone repair, and the enhancement of the PA/plasminogen system induced by inactivation of PAI-1 might compensate the bone repair process suppressed by the diabetic state. These findings are consistent with our previous findings that plasminogen deficiency impairs bone repair [23]. Further studies will be necessary to clarify the mechanisms, by which PAI-1 impairs bone repair.

We previously revealed that the induction of diabetes decreases bone mineral density (BMD) and bone strength index and suppresses the expression of osteogenic genes in female wild-type mice, but not in PAI-1-deficient mice [20]. In the present study, we revealed that PAI-1 deficiency blunted the decrease in the number of ALP-positive osteoblastic cells induced by the diabetic state. Moreover, the levels of osteogenic genes, such as Osterix, ALP, type I collagen, and osteocalcin, were decreased at the damaged site of femur in female diabetic wild-type mice. These effects seemed to be reversed by PAI-1 deletion. In addition, our previous study revealed that active PAI-1 treatment reduces the levels of Runx2, Osterix, and ALP mRNA as well as ALP activity and mineralization in primary osteoblasts obtained from female wild-type mice [20]. These findings suggest that PAI-1 deficiency alleviates the impairment of bone repair associated with the diabetic state partly by protecting against diabetes-impaired osteoblastogenesis at the damaged site.

PAI-1 might impair osteoblast function by directly affecting osteoblasts in the diabetic state in female mice during bone repair. Accumulating evidence suggests that the diabetic state impairs stem cell function and negatively regulates tissue repair [6], [11], [29]. Extrinsic signaling from the surrounding ECM plays a pivotal role in converting mesenchymal stem cells into commitment osteogenic lineage, then terminally differentiating into osteoblasts [30]. PAI-1 inhibits the function of PA, hence suppresses activations of pro-matrix metalloproteinases (MMPs), which finally interfere with ECM remodeling, in pathological conditions. Previous studies revealed that MMPs activity is increased in the fibrotic liver and asthmatic lung in PAI-1-deficient mice [31], [32]. Moreover, Krause *et al*. showed that the decrease in the levels of active MMP-9 in diabetic mice was restored by systemic treatment with a PAI-1 inhibitor, suggesting that the increase in PAI-1 levels induced by the diabetic state suppresses the activation of pro-MMPs [33]. Based on this knowledge, we can speculate that the protective effects of PAI-1 deficiency on the delayed bone repair induced by the diabetic state could be explained by the activation of MMPs, which modify the surrounding ECM microenvironments, consequently promoting mesenchymal stem cell differentiation into osteoblasts. Moreover, decreased growth factors such as insulin-like growth factor-I and fibroblast growth factor-2 are related to diminished bone formation during bone repair in diabetes [16]. The details of mechanism, by which PAI-1 is involved in the impaired bone repair process in the diabetic state, remain incompletely understood.

In the present study, PAI-1 deficiency significantly elevated the levels of Runx2, ALP, and type I collagen mRNA at the damaged site after a femoral bone defect in female mice without STZ treatment. Moreover, PAI-1 deficiency blunted the reduction in the number of ALP-positive cells induced by STZ treatment at the damaged site in mice. Our previous study showed that PAI-1 suppressed the levels of Runx2, ALP, and type I collagen mRNA in primary osteoblasts from mouse calvaria [20]. These findings suggest that PAI-1 suppresses osteoblast differentiation in both physiological and pathological states.

The fracture healing process can be divided into 3 stages: acute inflammation, repair, and remodeling. In the repair phase, the fracture site is repaired through the process of endochondral ossification, i.e., the formation of cartilage and the replacement of the cartilage by bone accompanied with the invasion of blood vessels [34]. Rundle *et al*. suggested that PAI-1 deficiency does not adversely affect the overall fracture healing process, but significantly enlarges the fracture callus in the non-diabetic state [35]. Our study showed that PAI-1 deficiency does not accelerate the bone repair process in female mice without diabetes, but it increases the cartilage matrix and the levels of chondrogenic marker mRNA such as type II collagen and aggrecan at the damaged site in female mice without diabetes. These results are consistent with the previous report by Rundle *et al*. [35]. However, their study showed that remodeling of the fracture callus was accelerated in PAI-1-deficient mice than in wild-type mice [35]. These data suggest enhancement of the tissue fibrinolytic system by PAI-1 deficiency that stimulates fracture healing through facilitation of ECM remodeling. The present study showed that PAI-1 deficiency rescued the impaired bone repair caused by the diabetic state. Taken together, PAI-1 might be involved in ECM remodeling during fracture healing in the diabetic state. Diabetes impairs cartilage formation during fracture healing through decreased chondrocyte differentiation and proliferation [17]. However, the diabetic state reduced the cartilage matrix and the levels of these chondrocyte markers in both wild-type and PAI-1-deficient mice in the present study, suggesting that PAI-1 deficiency could not compensate for the suppression of chondrogenesis induced by the diabetic state.

Mesenchymal stem cells have the ability to differentiate into chondrogenic and adipogenic lineage cells. Several studies revealed that an altered mesenchymal stem cell lineage selection toward adipocytes rather than osteoblasts is related to the mechanism of diabetic bone loss [6]. Bone marrow adiposity is observed in aged and type 1 diabetic bone tissues, which may be associated with a decrease in BMD [36]. Meanwhile, PAI-1 deficiency blunted the change in the levels of adipogenic genes that were increased with diabetes in tibia from female mice [20], suggesting that PAI-1 deficiency restores bone formation by promoting the differentiation of mesenchymal stem cells into osteoblasts instead of adipocytes. However, the present study revealed that the diabetic state did not induce PPAR-γ at the damaged site in wild-type female mice. Moreover, the levels of aP-2 mRNA were similar between diabetic wild-type and PAI-1-deficient mice, although the levels of aP-2 mRNA, known as an adipokine associated with the adipocyte phenotype, were increased in diabetic mice. These findings suggest that an altered mesenchymal stem cell lineage selection toward adipocytes rather than osteoblasts is not important for the bone repair process impaired by the diabetic state, compared to that in diabetic bone loss, because PPAR-γ is considered a key transcriptional factor that regulates adipogenesis [19], [20], [36].

In conclusion, our study showed that PAI-1 deficiency attenuates diabetic impaired bone repair in female mice partly through a reverse of the reduction in the number of osteoblasts induced by diabetic state, suggesting that PAI-1 is involved in the impaired bone repair induced by type I diabetes in female mice. Diabetic patients exhibit reduced BMD, an increase risk of fracture, and impairment in the fracture healing process. Our observations therefore provide a novel promising approach to ameliorate fracture healing impairment and promote bone regeneration in patients with diabetes.

## Supporting Information

Table S1 **Primers used for real-time PCR experiments.**
(DOC)

## References

[1] Janghorbani M, Van Dam RM, Willett WC, Hu FB (2007) Systematic review of type 1 and type 2 diabetes mellitus and risk of fracture. Am J Epidemiol 166: 495–505. 17575306 10.1093/aje/kwm106

[2] Vestergaard P (2007) Discrepancies in bone mineral density and fracture risk in patients with type 1 and type 2 diabetes—a meta-analysis. Osteoporos Int 18: 427–444. 17068657 10.1007/s00198-006-0253-4

[3] Hofbauer LC, Brueck CC, Singh SK, Dobnig H (2007) Osteoporosis in patients with diabetes mellitus. J Bone Miner Res 22: 1317–1328. 17501667 10.1359/jbmr.070510

[4] Merlotti D, Gennari L, Dotta F, Lauro D, Nuti R (2010) Mechanisms of impaired bone strength in type 1 and 2 diabetes. Nutr Metab Cardiovasc Dis 20: 683–690. 20934862 10.1016/j.numecd.2010.07.008

[5] Wongdee K, Charoenphandhu N (2011) Osteoporosis in diabetes mellitus: Possible cellular and molecular mechanisms. World J Diabetes 2: 41–48. 21537459 10.4239/wjd.v2.i3.41 PMC3083906

[6] McCabe LR (2007) Understanding the pathology and mechanisms of type I diabetic bone loss. J Cell Biochem 102: 1343–1357. 17975793 10.1002/jcb.21573

[7] Shin L, Peterson DA (2012) Impaired therapeutic capacity of autologous stem cells in a model of type 2 diabetes. Stem Cells Transl Med 1: 125–135. 23197759 10.5966/sctm.2012-0031 PMC3659680

[8] Liao YF, Chen LL, Zeng TS, Li YM, Fan Y, et al (2010) Number of circulating endothelial progenitor cells as a marker of vascular endothelial function for type 2 diabetes. Vasc Med 15: 279–285. 20511292 10.1177/1358863X10367537

[9] Shoji T, Koyama H, Morioka T, Tanaka S, Kizu A, et al (2006) Receptor for advanced glycation end products is involved in impaired angiogenic response in diabetes. Diabetes 55: 2245–2255. 16873687 10.2337/db05-1375

[10] Lerman OZ, Galiano RD, Armour M, Levine JP, Gurtner GC (2003) Cellular dysfunction in the diabetic fibroblast: impairment in migration, vascular endothelial growth factor production, and response to hypoxia. Am J Pathol 162: 303–312. 12507913 10.1016/S0002-9440(10)63821-7 PMC1851127

[11] Fadini GP, Albiero M, Vigili de Kreutzenberg S, Boscaro E, Cappellari R, et al (2013) Diabetes impairs stem cell and proangiogenic cell mobilization in humans. Diabetes Care 36: 943–949. 23111057 10.2337/dc12-1084 PMC3609511

[12] Simpson CM, Calori GM, Giannoudis PV (2012) Diabetes and fracture healing: the skeletal effects of diabetic drugs. Expert Opin Drug Saf 11: 215–220. 22145960 10.1517/14740338.2012.639359

[13] Kayal RA, Alblowi J, McKenzie E, Krothapalli N, Silkman L, et al (2009) Diabetes causes the accelerated loss of cartilage during fracture repair which is reversed by insulin treatment. Bone 44: 357–363. 19010456 10.1016/j.bone.2008.10.042 PMC2700945

[14] Kayal RA, Tsatsas D, Bauer MA, Allen B, Al-Sebaei MO, et al (2007) Diminished bone formation during diabetic fracture healing is related to the premature resorption of cartilage associated with increased osteoclast activity. J Bone Miner Res 22: 560–568. 17243865 10.1359/jbmr.070115 PMC3109431

[15] Kayal RA, Siqueira M, Alblowi J, McLean J, Krothapalli N, et al (2010) TNF-alpha mediates diabetes-enhanced chondrocyte apoptosis during fracture healing and stimulates chondrocyte apoptosis through FOXO1. J Bone Miner Res 25: 1604–1615. 20200974 10.1002/jbmr.59 PMC3154002

[16] Retzepi M, Donos N (2010) The effect of diabetes mellitus on osseous healing. Clin Oral Implants Res 21: 673–681. 20465554 10.1111/j.1600-0501.2010.01923.x

[17] Gandhi A, Beam HA, O'Connor JP, Parsons JR, Lin SS (2005) The effects of local insulin delivery on diabetic fracture healing. Bone 37: 482–490. 16027060 10.1016/j.bone.2005.04.039

[18] Mathieu P, Lemieux I, Despres JP (2010) Obesity, inflammation, and cardiovascular risk. Clin Pharmacol Ther 87: 407–416. 20200516 10.1038/clpt.2009.311

[19] Ma LJ, Mao SL, Taylor KL, Kanjanabuch T, Guan Y, et al (2004) Prevention of obesity and insulin resistance in mice lacking plasminogen activator inhibitor 1. Diabetes 53: 336–346. 14747283 10.2337/diabetes.53.2.336

[20] Tamura Y, Kawao N, Okada K, Yano M, Okumoto K, et al (2013) Plasminogen activator inhibitor-1 is involved in streptozotocin-induced bone loss in female mice. Diabetes 62: 3170–3179. 23715621 10.2337/db12-1552 PMC3749344

[21] Declerck PJ, Gils A (2013) Three decades of research on plasminogen activator inhibitor-1: a multifaceted serpin. Semin Thromb Hemost 39: 356–364. 23504606 10.1055/s-0033-1334487

[22] Daci E, Verstuyf A, Moermans K, Bouillon R, Carmeliet G (2000) Mice lacking the plasminogen activator inhibitor 1 are protected from trabecular bone loss induced by estrogen deficiency. J Bone Miner Res 15: 1510–1516. 10934649 10.1359/jbmr.2000.15.8.1510

[23] Kawao N, Tamura Y, Okumoto K, Yano M, Okada K, et al (2013) Plasminogen plays a crucial role in bone repair. J Bone Miner Res 28: 1561–1574. 23456978 10.1002/jbmr.1921

[24] Dempster DW, Compston JE, Drezner MK, Glorieux FH, Kanis JA, et al (2013) Standardized nomenclature, symbols, and units for bone histomorphometry: a 2012 update of the report of the ASBMR Histomorphometry Nomenclature Committee. J Bone Miner Res 28: 2–17. 23197339 10.1002/jbmr.1805 PMC3672237

[25] Pannekoek H, Veerman H, Lambers H, Diergaarde P, Verweij CL, et al (1986) Endothelial plasminogen activator inhibitor (PAI): a new member of the Serpin gene family. EMBO J 5: 2539–2544. 2430793 10.1002/j.1460-2075.1986.tb04532.x PMC1167150

[26] Sprengers ED, Kluft C (1987) Plasminogen activator inhibitors. Blood 69: 381–387. 3099859

[27] Allan EH, Martin TJ (1995) The plasminogen activator inhibitor system in bone cell function. Clin Orthop Relat Res: 54–63. 7641498

[28] Daci E, Udagawa N, Martin TJ, Bouillon R, Carmeliet G (1999) The role of the plasminogen system in bone resorption in vitro. J Bone Miner Res 14: 946–952. 10352103 10.1359/jbmr.1999.14.6.946

[29] Lu H, Kraut D, Gerstenfeld LC, Graves DT (2003) Diabetes interferes with the bone formation by affecting the expression of transcription factors that regulate osteoblast differentiation. Endocrinology 144: 346–352. 12488363 10.1210/en.2002-220072

[30] Stevens MM, George JH (2005) Exploring and engineering the cell surface interface. Science 310: 1135–1138. 16293749 10.1126/science.1106587

[31] Wang H, Zhang Y, Heuckeroth RO (2007) PAI-1 deficiency reduces liver fibrosis after bile duct ligation in mice through activation of tPA. FEBS Lett 581: 3098–3104. 17561000 10.1016/j.febslet.2007.05.049

[32] Oh CK, Ariue B, Alban RF, Shaw B, Cho SH (2002) PAI-1 promotes extracellular matrix deposition in the airways of a murine asthma model. Biochem Biophys Res Commun 294: 1155–1160. 12074598 10.1016/S0006-291X(02)00577-6

[33] Krause MP, Moradi J, Nissar AA, Riddell MC, Hawke TJ (2011) Inhibition of plasminogen activator inhibitor-1 restores skeletal muscle regeneration in untreated type 1 diabetic mice. Diabetes 60: 1964–1972. 21593201 10.2337/db11-0007 PMC3121432

[34] Claes L, Recknagel S, Ignatius A (2012) Fracture healing under healthy and inflammatory conditions. Nat Rev Rheumatol 8: 133–143. 22293759 10.1038/nrrheum.2012.1

[35] Rundle CH, Wang X, Wergedal JE, Mohan S, Lau KH (2008) Fracture healing in mice deficient in plasminogen activator inhibitor-1. Calcif Tissue Int 83: 276–284. 18820962 10.1007/s00223-008-9169-7

[36] Rosen CJ, Ackert-Bicknell C, Rodriguez JP, Pino AM (2009) Marrow fat and the bone microenvironment: developmental, functional, and pathological implications. Crit Rev Eukaryot Gene Expr 19: 109–124. 19392647 10.1615/critreveukargeneexpr.v19.i2.20 PMC2674609

