# Supplementary material for: Plasminogen Activator Inhibitor-1 Is Involved in Impaired Bone Repair Associated with Diabetes in Female Mice
Source: PLoS One. 2014 Mar 20;9(3):e92686. doi: 10.1371/journal.pone.0092686 (PMC3961397; doi:10.1371/journal.pone.0092686)
Supplement: Table S1 — Primers used for real-time PCR experiments. (DOC) [file pone.0092686.s001.doc]

**Table S1. Primers used for real-time PCR experiments.**

Gene Primer sequence

Runx2 Forward 5’-AAATGCCTCCGCTGTTATGAA-3’

Reverse 5’-GCTCCGGCCCACAAATCT-3’

Osterix Forward 5’-AGCGACCACTTGAGCAAACAT-3’

Reverse 5’-GCGGCTGATTGGCTTCTTCT-3’

ALP Forward 5’-ATCTTTGGTCTGGCTCCCATG-3’

Reverse 5’-TTTCCCGTTCACCGTCCAC-3’

Col I Forward 5’-GGTCAAAGGTTTGGAAGCAG-3’

Reverse 5’-TGTGAAATGCCACCTTTTGA-3’

Osteocalcin Forward 5’-CCTGAGTCTGACAAAGCCTTCA-3’

Reverse 5’-GCCGGAGTCTGTTCACTACCTT-3’

TRAP Forward 5’- GCAACATCCCCTGGTATGTG -3’

Reverse 5’- GCAAACGGTAGTAAGGGCTG -3’

RANKL Forward 5’- CACAGCGCTTCTCAGGAGCT -3’

Reverse 5’- CATCCAACCATGAGCCTTCC -3’

OPG Forward 5’- CTTGGGTCTGTTGCTTGGTGA -3’

Reverse 5’- GCCGCTTCCTTACACACCAG -3’

Col II Forward 5’-CCTCCGTCTACTGTCCACTGA-3’

Reverse 5’-ATTGGAGCCCTGGATGAGCA-3’

Col X Forward 5’-TGGGTAGGCCTGTATAAAGAACGG-3’

Reverse 5’-CATGGGAGCCACTAGGAATCCTGAGA-3’

Aggrecan Forward 5’- CCTGCTACTTCATCGACCCC-3’

Reverse 5’-AGATGCTGTTGACTCGAACCT -3’

PPAR-γ Forward 5’-GGAAAGACAACGGACAAATCAC-3’

Reverse 5’-TACGGATCGAAACTGGCAC-3’

aP-2 Forward 5’- ATCACCGCAGACGACAGGA -3’

Reverse 5’- CTCATGCCCTTTCATAAACT -3’

GAPDH Forward 5’-AGGTCGGTGTGAACGGATTTG-3’

Reverse 5’-GGGGTCGTTGATGGCAACA-3’

ALP = alkaline phosphatase; Col I = type I collagen; TRAP = tartrate-resistant acid phosphatase; RANKL = receptor activator of nuclear factor kB ligand; OPG = osteoprotegerin; Col II = type II collagen; Col X = type X collagen; PPAR-γ = peroxisome proliferator-activated receptor γ; aP-2 = adipocyte protein-2.
